# The Effects of High-Temperature Weather on Human Sleep Quality and Appetite

**DOI:** 10.3390/ijerph16020270

**Published:** 2019-01-18

**Authors:** Guozhong Zheng, Ke Li, Yajing Wang

**Affiliations:** School of Energy, Power and Mechanical Engineering, North China Electric Power University, Baoding 071003, China; like@ncepu.edu.cn (K.L.); wangyajing@ncepu.edu.cn (Y.W.)

**Keywords:** high-temperature weather, sleep quality, appetite, meal time, meal weight

## Abstract

High-temperature weather appears in high frequency, big strength, and long duration in the summer. It is therefore important to study the effects of high-temperature weather on sleep quality and appetite. Ten healthy college students were selected as subjects. The experiment conditions were divided by the daily maximum temperature into 28 °C, 32 °C, 36 °C, and 38 °C. The objective sleep quality was measured by an intelligent sleep monitoring belt, and the subjective sleep quality was measured by a questionnaire survey. The subjective appetites were assessed by a visual analog scale (VAS), and the objective appetites were assessed by the meal weight and the meal time. For sleep quality, the objective results indicated that the sleep quality at 32 °C was the best, followed by 28 °C, while the sleep quality at 36 °C and 38 °C was the worst. Significant effects were mainly reflected in sleep duration and shallow sleep. The subjective results showed that temperature had significant effects on sleep calmness, difficulty in falling asleep, sleep satisfaction, and sleep adequateness. For appetite, the VAS results indicated that high temperatures mainly led to a reduction of appetite at lunch time. The meal weights of lunch were larger than those of supper except for 28 °C, and the meal time of lunch and supper was longer than that of breakfast. The meal time of lunch was longer than that of supper except for 36 °C. This paper can provide a study method and reference data for the sleep quality and appetite of human in high-temperature weather.

## 1. Introduction

An average person spends about one third of their life sleeping. Adequate sleep is beneficial to restore physical strength and refresh the brain, and it helps to maintain good health and productivity of the human body. Nevertheless, insufficient sleep may disrupt the body’s cognitive function and immune function. For instance, sleep disorders can cause changes in immune mechanism-neuroendocrine-thermoregulation, and then result in a pathological process [1,2]. In recent years, high-temperature weather has shown the characteristics of high frequency, high intensity, and long duration [3,4]. To begin with, high temperatures cause thermal discomfort to the human body, even at night, and affect our quality of life [3,4,5]. In addition, high temperatures also suppress appetite. Food intake regulation is a mechanism of thermoregulation, and the feeding activity of human body may be affected by thermoregulatory response to prevent hyperthermia in a hot environment [6].

In actual life, a high-temperature environments is common when hot weather occurs [2]. In some places, such as school classrooms, student dormitories, security pavilion and remote places, air conditioners are not offered, people have to stay in these places for a long time and their sleep quality and appetite are adversely affected by high temperatures [5,6,7]. Furthermore, insufficient sleep may lead to frequent accidental injuries and huge economic losses [1], and the decreased appetite may cause weight loss or malnutrition, thus finally affecting health [8]. Therefore, to ensure physiological health, it is of great importance to study the effects of high-temperature weather on sleep quality and appetite.

Some scholars have studied the influence of temperature on the sleep quality of humans. Lan [9] selected eighteen students without sleep disorders as subjects, and the temperature conditions were set as 23 °C, 26 °C, and 30 °C in a sleep chamber. A subjective questionnaire, physiological parameters, and a measurement of work efficiency were adopted to evaluate sleep quality after getting up on the next day. The results indicated that sleep quality at 26 °C was the highest, and a higher or lower temperature would activate the body thermoregulation system and affect the subjects’ sleep quality. Pan [10] studied the thermal comfort of humans during sleep. The temperature conditions were divided into a summer group (23 °C, 26 °C, and 30 °C) and winter group (17 °C, 20 °C, and 23 °C). College students were selected as subjects. A subjective questionnaire was carried out and physiological parameters were measured. The results indicated that the temperature mainly affected the duration of sleep onset latency (SOL) and slow wave sleep (SWS). Okamoto et al. [11] examined the effects of mild heat exposure on sleep quality and the body temperature of elderly people. Ten healthy male elderly people were selected as subjects, and they were asked to sleep from 22:00 to 06:00. The environment conditions were set as: (1) 26 °C, 50% and (2) 32 °C, 50%. The electroencephalogram (EEG), electrooculography (EOG), mental electromyogram, rectal temperature, and skin temperature were continuously recorded. The tests indicated that, at 32 °C, the awakening rate and the total awakening time significantly increased, the duration of rapid eyes movement (REM) decreased, the fall in rectal temperature was significantly suppressed, and the mean skin temperature increased. Lan et al. [12] summarized that high temperatures resulted in decreased total sleep time, decreased duration of REM and SWS, and increased sleep onset latency (SOL) and wake after sleep onset (WASO). Zhu et al. [13] measured the environmental parameters of different bedrooms (including naturally ventilated rooms and air-conditioned rooms) for nearly one month during the summer in Beijing. Subjects’ sleep quality was monitored by Actiwatch 2 (Philips Respironics, Pittsburgh, Pennsylvania, USA). The results showed that in naturally ventilated rooms, temperature was significantly correlated to sleep efficiency, WASO, total sleep time, and number of awakenings. As shown in the above literature, the effects of temperature on sleep quality were reflected in several aspects. Firstly, the higher temperature would activate the body thermoregulation system and stress hormones were produced by central neuronal cells, then it resulted in increased wakefulness, and sleep quality was affected. Secondly, the SOL and SWS were affected by high temperatures, which caused a decreased duration of SWS and increased SOL. Lastly, the decrease of core temperature was significantly suppressed.

Some scholars have also conducted some studies on the appetite of humans. Wang [14] studied the effect of physical exercise on human appetite and food rations. Seven healthy males were selected as subjects, and they were asked to jog and swim under the same energy expenditure. Then, their oral temperatures were measured after 10 min of exercise. Half an hour after the exercise, their appetite and food intake were tested using an appetite scale (7 grades). The results showed that compared to swimming, the average body temperature increased and the appetite reduced in jogging condition. Westerterp-Plantenga et al. [15] assessed the effect of a short exposure to the thermoneutral temperature of 27 °C and the normal temperature of 22 °C on the energy metabolism in women. Eight healthy female volunteers participated in this study, and they were required to stay in the breathing room for 48 h. Then, they were fed in energy balance during the first 24 h, and fed ad libitum during the next 24 h. Their appetite was recorded by a visual analog scale (VAS), while their skin temperatures and core temperatures were measured continuously by thermistor probes. The results showed that compared with 22 °C, the respiratory quotient, the core temperature and the skin temperature all increased, while the differences between the rectal temperature and the distal temperature decreased. The reduction in energy intake was primarily related to the increase of body temperature. Geeraerts et al. [16] investigated the effect of experimentally induced anxiety on the gastric sensory–motor function of healthy people. In the experiment, the gastric sensitivity and accommodation ability of 14 subjects were evaluated by a gastric pressure instrument. Moreover, they were asked to carry out a full drinking test (30 mL/min) while epigastric symptoms were recorded by a VAS scale. The results indicated that anxiety led to a significant decrease in gastric compliance, and anxiety was significantly associated with a higher score of satiety, fullness, and satisfaction. To sum up, appetite was inhibited by high temperatures. On one hand, high temperatures led to a high core body temperature, then the increased core body temperature caused poor appetite and reduced energy intake. On the other hand, anxiety was associated with high temperatures and led to a higher fullness and satisfaction.

In this study, sleep quality and human appetite were adopted to study the quality of life in high-temperature weather. Ten key sleep quality parameters were adopted to evaluate objective sleep quality, and a combined subjective questionnaire was used to comprehensively evaluate subjective sleep quality. A VAS scale was used to assess the subjective appetite, and the meal weight and meal time were adopted to assess the objective appetite.

This study mainly differs from previous studies in the following aspects: (1) providing both an objective method and a subjective method to evaluate sleep quality and appetite; (2) adopting meal weight and meal time to assess objective appetite; (3) adding food grease into a new VAS scale to measure the degree of greasiness of the food.

## 2. Materials and Methods

### 2.1. Subjects

A total of ten college students, including five males and five females, were selected as subjects. The subjects should be in good health condition and have no diseases, such as hypertension, sleep disorders, and so on. During the experiment, all subjects were not allowed to drink coffee, drink alcohol, and take drugs. Table 1 shows the anthropometric information of the subjects.

The sample size of the subjects was considered and determined as follows: (1) The sample sizes of related literature on human experiments varied from 7 to 14 [17,18,19,20,21], thus ten subjects were initially selected for this study; (2) the statistical power of the sample size (*n* = 10) was calculated by the PASS software (NCSS, Kasville, Utah, USA) to show the power of statistical analyses. The results indicated that the statistical power value of the paired sample *t* test of ten subjects was 0.940. This shows that 10 subjects would provide sufficient statistical power to detect a difference.

All participants provided written informed consent, and they were informed of the study purpose. The study was approved by the Chinese Ethics Committee of Registering Clinical Trials (No. ChiECRCT-20170108) and conformed to the principles outlined in the Declaration of Helsinki.

### 2.2. Experiment Conditions

There was a positive correlation between temperature and heat-related diseases [22,23,24,25,26]; therefore, the daily maximum temperature displayed more strength in reflecting the effects of the environment intensity on the human body, therefore it provided a more spatially robust measure of heat stress. Thus, in this study, the experiment conditions were divided by the daily maximum hourly outdoor temperature, namely 28 °C, 32 °C, 36 °C, and 38 °C (the maximum hourly outdoor temperature ± 1.0 °C), respectively. Each temperature condition represents a high-temperature day. The experiments were conducted in a top floor non-air-conditioned house in Baoding, and the house maintained natural ventilation during the experiment.

**Figure 1:**
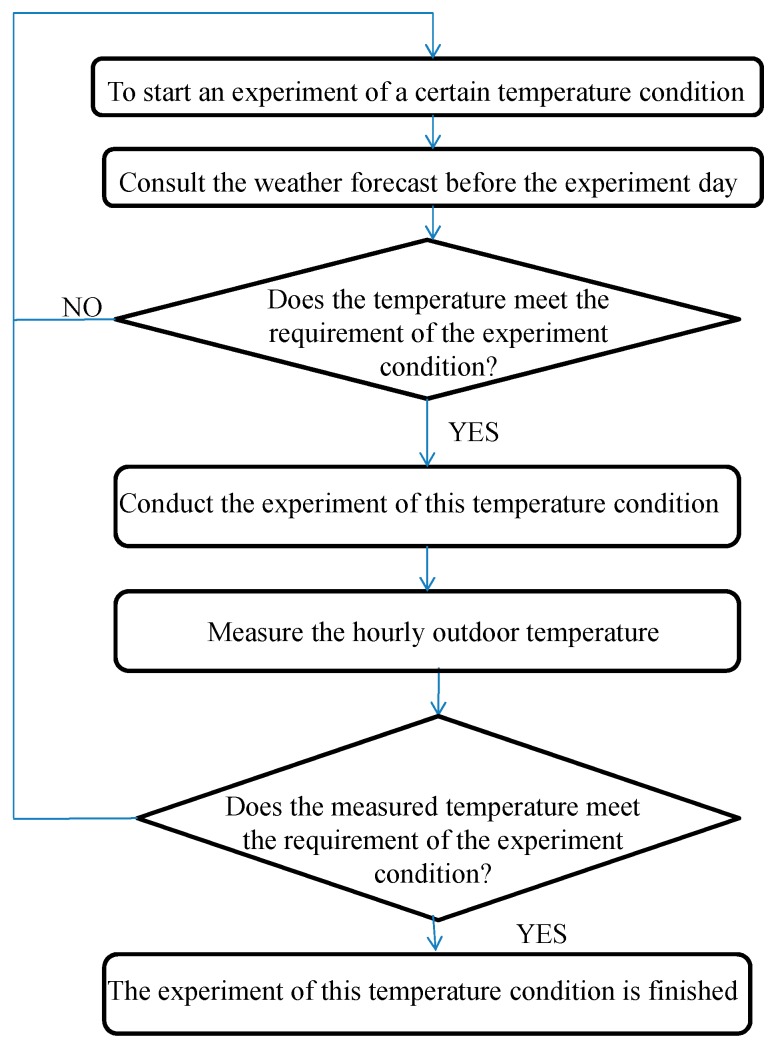
Flowchart to select an experiment day of a certain temperature condition.

Most experiments of related literature were conducted in an experiment chamber. In this study, the experiment was conducted in an actual site. The experiment chamber can supply ideal temperature control; however, the space in the chamber is closed and narrow, and it cannot supply natural daylight. In this study, the experiment duration of each temperature condition lasted for 24 h. If the experiments are conducted in an experimental chamber, there may be issues of psychological anxiety, fuzziness, impatience, and other abnormal emotions, and finally influence the experiment results. For actual site, although the temperature is affected by the weather, if the experiment day is well-selected, the above problems can be avoided. In this study, all experiments in different temperature conditions were conducted on sunny days in the summer. Although the maximum temperatures were different, the changing trends of the outdoor temperature were similar. To choose the experiment day of a certain temperature condition, the hourly temperature of the weather forecast before the experiment day was consulted. When that met the experiment condition, the experiment was then conducted in the next day. When conducting the experiment, the hourly outdoor temperature of the experiment day was also measured to verify the experiment condition. When the measured hourly outdoor temperature met the requirement of the experiment condition, the experiment condition was finished. Otherwise, the experiment of this temperament condition was to be reconducted. The flowchart to select an experiment day of a certain temperature condition is shown in Figure 1.

### 2.3. Experiment Instruments

The parameters and instruments in the experiment are summarized in Table 2.

### 2.4. Sleep Quality

In the experiments, a monitoring belt was used to measure objective sleep quality. It is a nonwearable intelligent sleep monitoring belt which is laid on the mattress. It can monitor some physiological parameters, such as heart rate, respiratory rate, turn over, and sleep cycle in real time. Then, the sleep quality data can be obtained from the software of Sleepace. Ten key objective evaluation indices, including sleep latency, actual sleep time, total sleep time, awakening frequency, deep sleep, moderate sleep, light sleep, awake phase, average heart rate, and average respiratory rate, can be obtained. When using the monitoring belt, subjects do not need to change their sleep habits.

The sleep questionnaire [27] was adopted to measure the subjective sleep quality in this paper. The questionnaire is shown in Table 3. The qualitative features of sleep were characterized by items 1 to 6, which included calmness of sleep, ease of falling asleep, ease of awakening, freshness after awakening, satisfaction about sleep, and sufficient sleep [27]. Items 7 to 9 characterized the quantitative features of sleep, which included time required for falling asleep, frequency of waking at night, and duration of each waking up. All items used 5 points except for item 6, which used a two-level score. For all items, the higher the level is, the better the sleep quality is [10,27].

### 2.5. Appetite

In the experiment, a subjective index and objective index for appetite measurement were both considered. For subjective parameters, a visual analog scale (VAS) was used to quantify a subject’s feelings on desire to eat, hunger, prospective consumption, and fullness [28,29]. To measure the greasiness degree of the food, food greasiness was added in the VAS. The scale 0–10 was adopted to evaluate the sensation before or after eating, and the higher the scale, the stronger the sensation. Based on their experience, the subjects were free to calibrate on each line that best matched how they were feeling at the time. The VAS questionnaire is shown in Table 4. Among these items, the feeling of fullness was evaluated after the meals, while the other items were evaluated before the meals. 

For objective measurement, the meal weight and the meal time were adopted. The meal weight was measured by an electronic scale before and after meals. The duration used for the whole meal was recorded to measure the meal time. At all temperature conditions, breakfast contained milk, eggs, and bread, lunch contained rice and a fried dish, and supper contained noodles and sausages. Thus, the caloric content of the three meals was constant under the four temperature conditions. In addition, all of the meals were provided in advance.

### 2.6. Experiment Process

For each experiment day, subjects were asked to arrive at the experiment house at 08:30, then rest quietly for 30 min to calm down their physiological state. The experiment continued from 09:00 to 09:00 on the next day. Furthermore, the intervals between each temperature condition lasted at least four days, and all subjects’ sleep and appetite were recorded at the same experiment house on all temperature conditions. During the experiment period, subjects were required to stay in the house, and they could rest or engage in mental activities, such as reading or writing.

When measuring sleep quality, bamboo mats were used. All subjects could freely add or decrease the number of their clothes and quilts. The subjects could choose their bedtime according to their sleep habits. Before bedtime, subjects were asked to lay the monitoring belt on the mattress near their chest and abdomen. Then, the monitor was turned on and the parameters were recorded. After getting up in the next morning, the monitor was turned off. In addition, the subjects were asked to fill out the sleep quality questionnaire.

To measure appetite, the subjects were asked to have breakfast, lunch, and supper at 08:00, 12:30, and 18:30 separately. Before each meal, the subjects were asked to fill out the VAS questionnaire. Before and after the meals, the weights of the foods were measured, and the meal weights were obtained from the weight differences. Meanwhile, the time used for the whole meal was recorded to measure the meal time.

**Figure 2:**
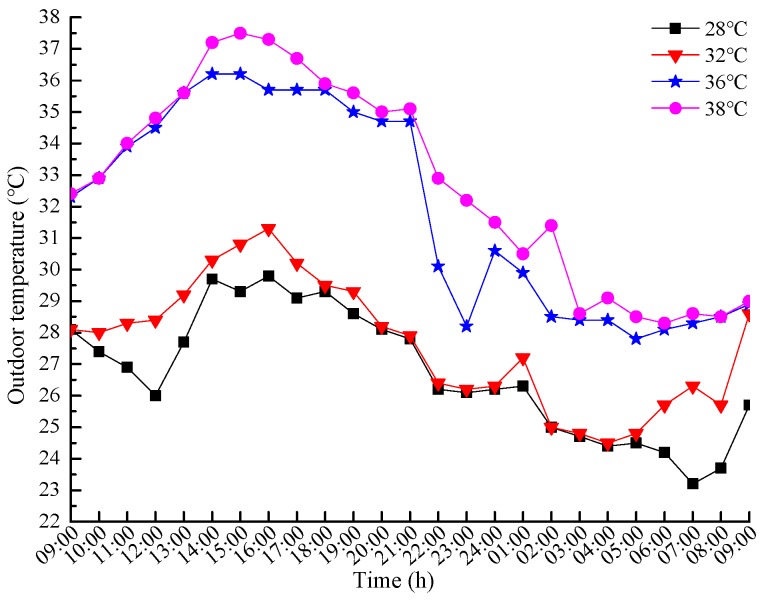
The hourly outdoor temperature of the four temperature conditions.

In addition, during each experiment group of a certain temperature condition, the hourly outdoor temperatures were measured every hour in the continuous 24 h. The results are shown in Figure 2.

### 2.7. Statistical Method

Since the sample size was less than 50, the normal tests of the measuring data among temperature conditions were conducted using the Shapiro-Wilk test. The test rejects the hypothesis of normality when the *p*-value is less than or equal to 0.05. When comparing the measuring data among temperature conditions, if the two sets of data were normally distributed, the paired sample *t* test was used. If the two sets of data did not follow normal distribution, then a two-pair sample test in nonparametric tests should be used. *p*-values less than 0.05 were considered statistically significant. In this paper, six paired of tests were done, including 28–32 °C, 28–36 °C, 28–38 °C, 32–36 °C, 32–38 °C, and 36–38 °C.

## 3. Results

### 3.1. The Effects of Temperature on Sleep Quality

**Figure 3:**
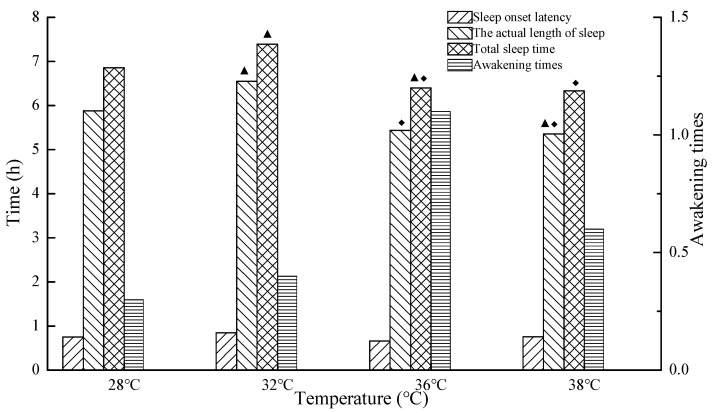
Sleep onset latency, actual length of sleep, total sleep time, and awakening time at 28 °C, 32 °C, 36 °C, and 38 °C (The legend ▲ indicates that there is a significant difference compared with 28 °C (*p* < 0.05), the legend ◆ indicates that there is a significant difference compared with 32 °C (*p* < 0.05)).

**Figure 4:**
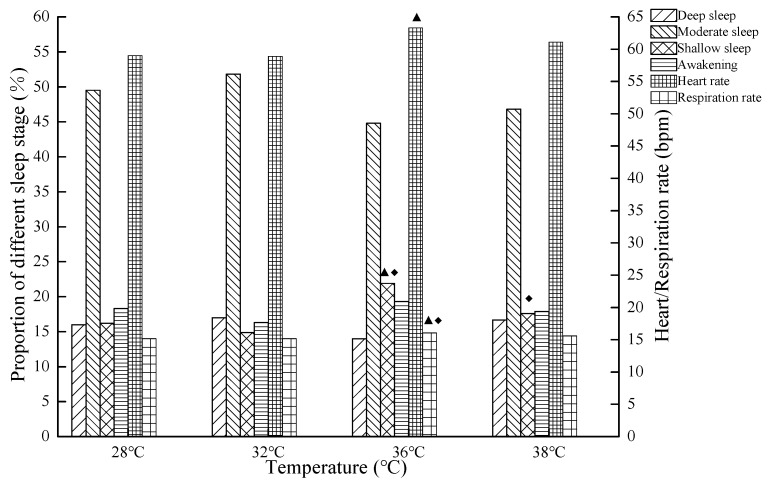
The average heart rate, average respiration rate, and proportion of deep sleep, moderate sleep, shallow sleep, and awakening, at 28 °C, 32 °C, 36 °C, and 38 °C (The legend ▲ indicates that there is a significant difference compared with 28 °C (*p* < 0.05), the legend ◆ indicates that there is a significant difference compared with 32 °C (*p* < 0.05)).

The relationships of objective assessment of sleep quality and temperature are shown in Figure 3 and Figure 4. The actual sleep time, total sleep time, shallow sleep, average heart rate, and average respiration rate were influenced by temperature. Furthermore, the actual sleep time and total sleep time at 32 °C were significantly longer than those at 28 °C (*p* < 0.05); the actual sleep time and total sleep time at 36 °C were significantly shorter than those at 32 °C (*p* < 0.05); and the total sleep time at 36 °C were significantly shorter than those at 28 °C (*p* < 0.05). The average heart rate at 36 °C was significantly larger than that at 28 °C (*p* < 0.05), and the shallow sleep and average respiration rate at 36 °C were significantly larger than those at 28 °C and 32 °C (*p* < 0.05). The actual sleep time at 38 °C was significantly shorter than that at 28 °C and 32 °C, while the total sleep time at 38 °C was significantly shorter than that at 32 °C (*p* < 0.05). The shallow sleep at 38 °C was significantly larger than that at 32 °C (*p* < 0.05). However, there was no significant difference between 36 °C and 38 °C (*p* < 0.05). The results indicate that sleep quality at 32 °C was the best, followed by 28 °C, and that at 36 °C and 38 °C was the worst. The significant effects of high temperatures on sleep quality were mainly reflected in the duration of sleep and shallow sleep. 

**Figure 5:**
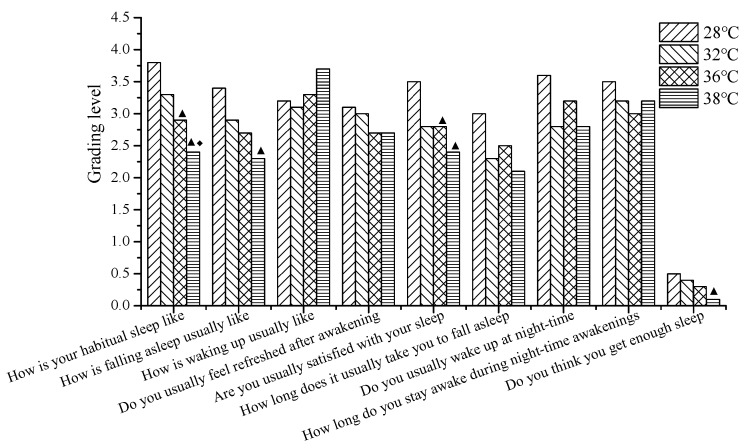
The subjective evaluation results of sleep quality at 28 °C, 32 °C, 36 °C, and 38 °C (The legend ▲ indicates that there is a significant difference compared with 28 °C (*p* < 0.05), the legend ◆ indicates that there is a significant difference compared with 32 °C (*p* < 0.05)).

The relationships of subjective assessment of sleep quality and temperature are shown in Figure 5. The calmness of sleep, ease of falling asleep, satisfaction about sleep, and sufficient sleep were influenced by temperature. Furthermore, the calmness of sleep and satisfaction about sleep at 36 °C were significantly lower than those at 28 °C (*p* < 0.05); the calmness of sleep, the ease of falling asleep, the satisfaction about sleep, and sufficient sleep at 38 °C were significantly lower than those at 28 °C (*p* < 0.05), and the calmness of sleep at 38 °C was significantly lower than that at 32 °C (*p* < 0.05). To sum up, the subjective evaluation on sleep quality significantly reduced, especially in the higher temperatures.

### 3.2. The Effects of Temperature on Appetite

The relationships of objective appetite and temperature are shown in Table 5. At the four temperature conditions, the meal weights of the breakfast were 389.8–447.2 g, and there was little difference among the four temperature conditions. The meal weights of the lunch were 507.7–614.6 g, while the meal weights of the supper were 471.4–570.9 g, which indicates that the meal weights of lunch were larger than those of supper except for 28 °C. The meal time of breakfast was 8.5–11.6 min, and there was also little difference among the four temperature conditions. The meal time of lunch and supper was longer than that of breakfast, and the meal time of lunch was longer than that of supper except for 36 °C. It was 10.6–19.8 min at lunch, while it was 12.6–15.7 min at supper, and there was little difference among the four temperature conditions. 

The relationships of subjective appetite and temperature are shown in Table 6. Subjects’ appetite for lunch was significantly affected by temperature. The desire to eat, hunger, and prospective consumption at 36 °C were significantly lower than those at 32 °C. Fullness at 38 °C was significantly lower than that at 32 °C, and fullness at 32 °C was significantly higher than that at 28 °C. Food greasiness at 38 °C and 36 °C was lower than that at 32 °C, and food greasiness at 32 °C was higher than that at 28 °C. The fullness of supper at 38 °C was significantly lower than that at 32 °C. This indicates that the subjective appetite for lunch at 32 °C was the best. This result is similar to that for objective sleep quality.

To sum up, the effects of temperature on appetite were mainly reflected in subjective appetite at lunch, and there was no significant effect on breakfast and supper. 

## 4. Discussion

The timing factors of natural environment (such as light and temperature) had a simultaneous impact on human sleep. The sleep-wake rhythm was also affected by the circadian rhythm of core body temperature [30]. However, during actual sleep, the temperature was not constant; thus, sleep quality was also affected by the variable temperature, and the changes of the set-point of body temperature were affected by the temperature fluctuation [31,32]. The consistency of body temperature and sleep rhythm suggests that when body temperature is maintained at a high level at night time, the body’s deep sleep time is reduced [13]. Secondly, the decrease of core temperature during sleep is more associated with an increase in peripheral skin dissipation, and better sleep quality is caused by increased peripheral heat loss and/or core reduction [33,34]. While the human body is exposed to high temperatures at night, the temperature gradient between the peripheral skin temperature and the environment is reduced; thus, the heat transfer from skin to environment decreases. Therefore, active vasodilation and wakefulness increases [11,35], and the decline of the core temperature slows down. In addition, the increase of human metabolism also results in the increase of oxygen consumption and the core body temperature [13]. Moreover, due to the little adjustment of quilt and pajamas in summer, SWS is closely related to high temperatures, and it mainly influences the first half step of sleep before midnight [34]. To sum up, body temperature regulation would affect sleep quality, and due to the decrease of nocturnal fall in body temperature, the minimum of rectal temperature increases, and the heat loss of peripheral skin is reduced, and finally, wakefulness increases and the SWS decreases.

In this study, subjective questionnaires showed that at 36 °C and 38 °C, it was difficult to fall asleep, and both sleep satisfaction and sufficiency were low, which indicates that high temperatures led to poor sleep quality. Combined with the objective parameters of sleep quality, it was further found that sleep quality at 32 °C was better than that at 28 °C. The reason is that at a lower temperature, the activity of thermoregulation nerve center increased, resulting in the increase of stress hormone secretion and wakefulness [24,36].

In the present study, the average heart rate and respiratory rate in the sleep stage significantly increased, and the average heart rate and respiratory rate of the 36 °C group was higher than that of 32 °C and 28 °C. High temperatures result in poor sleep quality, thermal discomfort, and an increase of the heart rate [10]. During SWS, blood pressure decreased, the heart rate slowed down, and breathing became smoother [30]. However, the heat stimulation resulted in increasing wakefulness and a shortened SWS duration [34]. Therefore, high temperatures caused an increase of the average heart rate and the respiratory rate during sleep.

In terms of appetite, for physiological factors, heat load in the body would affect energy and water metabolism, and then affect the appetite and digestive function of the human body [37]. High temperatures resulted in the increase of the core temperature, and then the digestive enzyme activity and metabolic rate declined [8,14]; thus, appetite was affected. In addition, a large amount of water diluted the gastric juice, leading to a decrease of acidity and loss of appetite [38]. For psychological factors, gastric motility was a key mediator of hunger, satiation, and satiety, and psychological factors played a causal role in the pathogenesis of some dyspeptic symptoms [16,39]. High temperatures may also cause an increment of the negative emotions [40]. Thus, the gastric sensor and motor function influenced the hunger and satiety responses, and finally appetite was reduced. In this study, subjects’ appetite for lunch was significantly affected by the temperature. Their desire to eat, hunger, prospective consumption, and food greasiness before lunch was reduced; meanwhile, the fullness at the end of the meal was obviously reduced. However, subjects’ appetite at breakfast and supper time was not significantly affected by the temperature; the reason is that the temperature at breakfast and supper time was significantly lower than that at lunch time.

The strengths of this study lie in: (1) introducing both of the subjective and objective indices to assess sleep quality and appetite from different points of view; (2) adding food greasiness into a new VAS scale to measure the greasiness degree of the food; (3) introducing the meal weight and meal time to assess objective appetite.

The shortcoming of this study lies in the sample size and sample source. Firstly, although the statistical power of the sample size showed that ten subjects would provide sufficient statistical power to detect a difference, a larger sample size will show larger power and greater strength in the accuracy and applicability of the results. Secondly, the subjects in the experiments were only composed of college students. There are some differences in the age and the physical quality between college students and the people in other occupations. Subjects from more job backgrounds will enhance the commonality of the results. Therefore, in further studies, subjects from different job backgrounds can be selected and a larger sample size can be considered.

## 5. Conclusions

Sleep quality at 32 °C was the best, followed by that at 28 °C, while sleep quality at 36 °C and 38 °C was the worst. The significant effects of high temperatures on sleep quality were mainly reflected in sleep duration and shallow sleep.

Temperature had significant effects on sleep calmness, difficulty in falling asleep, sleep satisfaction, and sleep adequateness. The effects of temperature on these four indicators were significant.

High temperatures led to a decrease in body temperature and an increase of the average heart rate and respiratory rate. Thus, wakefulness increased and SWS decreased.

For subjective appetite, effects were mainly reflected in appetite at lunch, and there was no significant effect on breakfast and supper. Subjective appetite for lunch at 32 °C was the best. Hunger and the desire to eat before lunch at 36 °C were significantly lower than those at 32 °C, and food greasiness before lunch at 38 °C and 36 °C was lower than that at 32 °C. 

For objective appetite, the meal weights of lunch were larger than those of supper, except for 28 °C, and the meal time of lunch and supper was longer than that of breakfast. The meal time of lunch was longer than that of supper except for 36 °C.

To sum up, this study analyzed the adverse effects of high temperatures on the human quality of life in two aspects: (1) poor sleep quality; (2) loss of appetite. This can provide methods to evaluate and measure sleep quality and appetite for actual conditions. It can also provide quantitative basic data for the changes in the sleep quality and appetite of the human body in high-temperature weather and give early warning information for the health-disadvantaged to protect their health.

## Figures and Tables

**Table 1:** Anthropometric information of subjects.

Gender	Age (years old)	Height (cm)	Weight (kg)	BMI *^a^* (kg/m^2^)
Male	22.4 ± 1.7	172.2 ± 5.1	69.2 ± 12.2	23.2 ± 2.9
Female	23.0 ± 1.4	161.4 ± 4.7	53.8 ± 8.1	20.6 ± 2.8

*^a^* Body mass index; BMI = weight/height^2^, normally between 18 and 25 kg/m^2^.

**Table 2:** Parameters and instruments.

Parameter	Instrument	Model	Precision
Outdoor temperature	Thermal index meter	AZ8778	±0.6 °C
Objective sleep quality	Sleep monitoring belt	Z101	/
Subjective sleep quality	Sleep questionnaire	/	±0.1 °C
Meal weight	Electronic scale	EK813	±1 g
Meal time	Watch	/	±1 min
Subjective appetite	VAS scale	/	/

**Table 3:** Items of the sleep quality questionnaire.

Questions	Score					
	5	4	3	2	1	0
How is your habitual sleep like?	Very calm	Fairly calm	Neither calm nor restless	Quite restless	Very restless	/
How is falling asleep usually like?	Very easy	Fairly easy	Neither easy nor difficult	Quite difficult	Very difficult	/
How is waking up usually like?	Very easy	Fairly easy	Neither easy nor difficult	Quite difficult	Very difficult	/
Do you usually feel refreshed after awakening?	Fully	Fairly	Moderately	Not much	Not at all	/
Are you usually satisfied with your sleep?	Fully	Fairly	Moderately	Not much	Not at all	/
Do you think you get enough sleep?	/	/	/	/	Yes	No
How long does it usually take you to fall asleep?	<5 min	5–10 min	10–20 min	20–30 min	>30 min	/
Do you usually wake up at night-time?	Never	Seldom	Sometimes	Often	Always	/
How long do you stay awake during night-time awakenings?	0	<5 min	5–15 min	15–30 min	30–60 min	/

**Table 4:** The VAS questionnaire.

Item	VAS Scale
Desire to eat	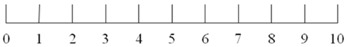
Hunger	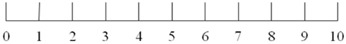
Prospective consumption	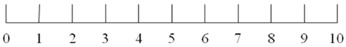
Fullness	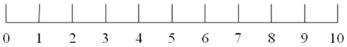
Food greasy	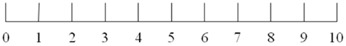

**Table 5:** The objective appetite in the four temperature conditions.

Index	Meal	Objective Appetite			
		28 °C	32 °C	36 °C	38 °C
Meal weight (g)	Breakfast	398.9 ± 170.1	389.8 ± 160.5	447.2 ± 182.4	389.8 ± 153.5
	Lunch	529.1 ± 178.6	614.6 ± 240.1	507.7 ± 225.1	568.2 ± 146.9
	Supper	570.9 ± 321.2	567.8 ± 266.9	471.4 ± 159.9	530.7 ± 165.9
Meal time (min)	Breakfast	9.4 ± 4.2	10.9 ± 2.9	11.6 ± 4.3	8.5 ± 4.2
	Lunch	13.3 ± 8.2	19.8 ± 7.9	10.6 ± 3.9	16.9 ± 3.9
	Supper	12.6 ± 3.2	14.9 ± 6.8	15.7 ± 5.4	14.1 ± 5.5

**Table 6:** The subjective appetite in the four temperature conditions.

Index	Meal	Subjective Appetite			
		28 °C	32 °C	36 °C	38 °C
Desire to eat	Breakfast	4.0 ± 2.3	4.4 ± 1.8	5.3 ± 2.1	4.8 ± 1.8
	Lunch	5.0 ± 1.6	5.5 ± 0.9	3.7 ± 1.5 ^◆^	4.8 ± 1.8
	Supper	5.7 ± 1.2	5.6 ± 1.3	6.1 ± 1.7	5.8 ± 1.8
Hunger	Breakfast	4.7 ± 2.6	4.4 ± 0.9	5.3 ± 1.9	4.4 ± 1.2
	Lunch	4.8 ± 1.8	5.4 ± 1.2	3.2 ± 1.8 ^◆^	5.1 ± 2.1
	Supper	5.6 ± 1.1	5.4 ± 1.6	6.0 ± 1.4	6.2 ± 1.5
Prospective consumption	Breakfast	4.2 ± 2.6	4.4 ± 1.1	5.6 ± 2.4	4.7 ± 1.6
	Lunch	5.5 ± 2.1	5.9 ± 1.3	3.7 ± 1.8 ^◆^	5.4 ± 2.3
	Supper	5.9 ± 1.4	5.4 ± 1.4	5.6 ± 1.4	6.2 ± 1.6
Fullness	Breakfast	5.0 ± 1.6	4.6 ± 1.7	4.6 ± 2.1	5.0 ± 1.9
	Lunch	5.3 ± 1.7	6.8 ± 1.9 ^▲^	5.7 ± 0.8	5.1 ± 1.6 ^◆^
	Supper	5.5 ± 1.4	6.9 ± 1.8	6.4 ± 1.4	5.6 ± 1.8 ^◆^
Food greasy	Breakfast	2.5 ± 2.5	3.9 ± 1.6	4.9 ± 2.1	4.8 ± 2.7
	Lunch	5.5 ± 1.1	7.7 ± 2.0 ^▲^	5.6 ± 0.9 ^◆^	4.8 ± 2.5 ^◆^
	Supper	5.9 ± 1.6	5.8 ± 1.5	5.1 ± 1.2	5.5 ± 1.3

▲ indicates that there is a significant difference compared with 28 °C (*p* < 0.05); ◆ indicates that there is a significant difference compared with 32 °C (*p* < 0.05).
